# The genome sequence of wild privet, *Ligustrum vulgare* L.

**DOI:** 10.12688/wellcomeopenres.24023.1

**Published:** 2025-05-16

**Authors:** Zoë A. Goodwin, David Bell, Vladimir Krivtsov, Michelle L. Hart, Royal Botanic Garden Edinburgh Genome Acquisition Lab, Darwin Tree of Life Barcoding collective, Plant Genome Sizing collective, Wellcome Sanger Institute Tree of Life Management, Samples and Laboratory team, Wellcome Sanger Institute Scientific Operations: Sequencing Operations, Wellcome Sanger Institute Tree of Life Core Informatics team, Tree of Life Core Informatics collective, Darwin Tree of Life Consortium

**Affiliations:** 1 Royal Botanic Garden Edinburgh, Edinburgh, Scotland, UK

**Keywords:** Ligustrum vulgare, wild privet, genome sequence, chromosomal, Lamiales

## Abstract

We present a genome assembly from a specimen of *Ligustrum vulgare* (wild privet; Streptophyta; Magnoliopsida; Lamiales; Oleaceae). The genome sequence has a total length of 1,384.00 megabases. Most of the assembly is scaffolded into 23 chromosomal pseudomolecules. The multipartite mitochondrial genome consists of two circularised molecules with lengths of 844.62 and 118.10 kilobases, and the plastid genome assembly is 161.82 kilobases long. Gene annotation of this assembly on Ensembl identified 31,482 protein-coding genes.

## Species taxonomy

Eukaryota; Viridiplantae; Streptophyta; Streptophytina; Embryophyta; Tracheophyta; Euphyllophyta; Spermatophyta; Magnoliopsida; Mesangiospermae; eudicotyledons; Gunneridae; Pentapetalae; asterids; lamiids; Lamiales; Oleaceae; Oleeae; *Ligustrum*; *Ligustrum vulgare* L. (NCBI:txid13597).

## Background

The wild privet, *Ligustrum vulgare* L. *,* is a deciduous to semi-evergreen shrub native to Britain and the Channel Islands, and a neophyte in Ireland ( Stroh *et al.*, 2023). It has a largely European distribution, occurring from Scandinavia to North Africa, and east to Iran ( WFO, 2025).

It is commonly found in hedgerows, woodland, and scrub, preferring well-drained calcareous soils ( Stroh *et al.*, 2023). With insect-pollinated, fragrant, small white flowers from June to August, wild privet is an important food source for many insects. These include the enormous caterpillars of the privet hawk moth *Sphinx ligustri* ( Waring *et al.*, 2017). The black berries are eaten and dispersed by birds ( Snow & Snow, 1988), though all parts of the plant are toxic to humans ( Cooper & Johnson, 1998). Various parts of *Ligustrum vulgare* are demonstrated to have medicinal potential, including strong antiproliferative properties for the treatment of cancer ( Litewski *et al.*, 2024). Like the more reliably evergreen privet, *Ligustrum ovalifolium* Hassk., wild privet is commonly used in garden hedging.

Here we present a chromosomally complete genome sequence for *Ligustrum vulgare*, based on a plant growing at the Royal Botanic Garden Edinburgh (Inverleith), Midlothian, Scotland, UK that was originally collected from Epsom Downs, Surrey. The assembled genome presented here is consistent with published chromosome counts of 2 *n* = 46 ( BSBI, 2025).

## Genome sequence report

**Figure 1.**
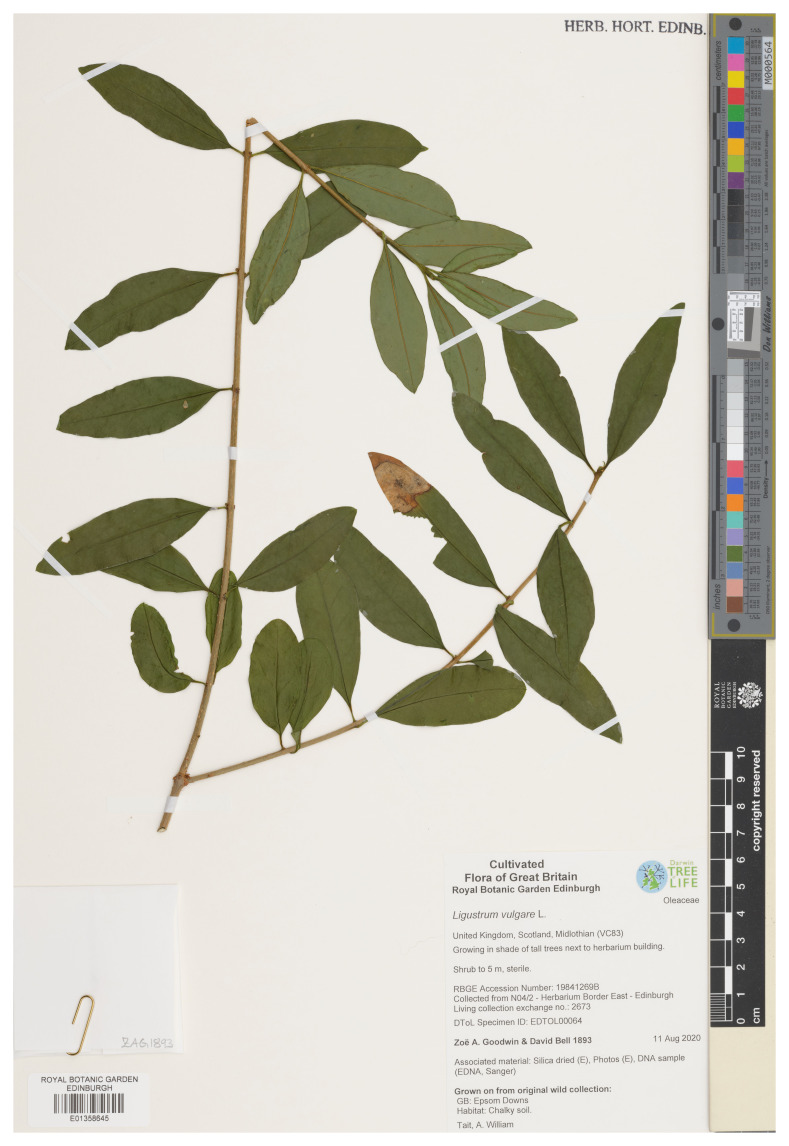
Photograph of the *Ligustrum vulgare* (daLigVulg1) specimen used for genome sequencing.

The genome size (1C-value) of the *Ligustrum vulgare* specimen ( Figure 1) was estimated to be 1.71 pg, equivalent to 1,670 Mb, by flow cytometry. The genome was sequenced using Pacific Biosciences single-molecule HiFi long reads, generating a total of 6.18 Gb (gigabases) from 0.45 million reads, providing approximately 34-fold coverage. Primary assembly contigs were scaffolded with chromosome conformation Hi-C data, which produced 176.58 Gb from 1,169.42 million reads. Specimen and sequencing details are provided in Table 1.

**Table 1.** Specimen and sequencing data for *Ligustrum vulgare*.

Project information			
**Study title**	Ligustrum vulgare (common privet)		
**Umbrella BioProject**	PRJEB65698		
**Species**	*Ligustrum vulgare*		
**BioSample**	SAMEA7535976		
**NCBI taxonomy ID**	13597		
Specimen information			
**Technology**	**ToLID**	**BioSample accession**	**Organism part**
**PacBio long read sequencing**	daLigVulg1	SAMEA8596958	leaf
**Hi-C sequencing**	daLigVulg1	SAMEA111431662	leaf
**RNA sequencing**	daLigVulg1	SAMEA111431662	leaf
Sequencing information			
**Platform**	**Run accession**	**Read count**	**Base count (Gb)**
**Hi-C Illumina NovaSeq 6000**	ERR12035282	1.17e+09	176.58
**PacBio Sequel IIe**	ERR12015761	1.78e+06	26.54
**PacBio Sequel IIe**	ERR12015762	1.61e+06	24.23
**PacBio Sequel IIe**	ERR12015763	4.49e+05	6.18
**RNA Illumina NovaSeq 6000**	ERR12035283	6.83e+07	10.31

**Figure 2.**
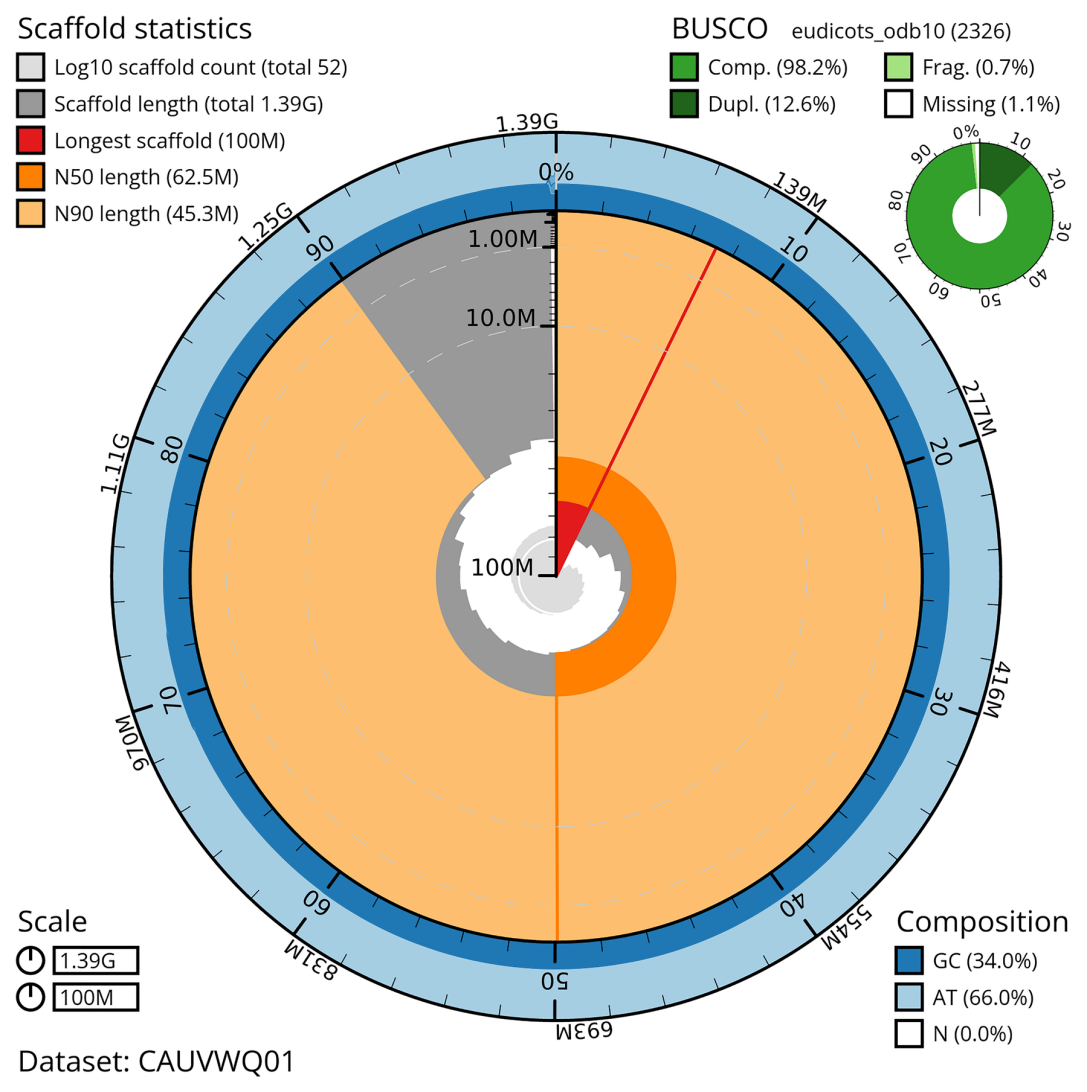
Genome assembly of *Ligustrum vulgare*, daLigVulg1.1: metrics. The BlobToolKit snail plot shows N50 metrics and BUSCO gene completeness. The main plot is divided into 1,000 size-ordered bins around the circumference with each bin representing 0.1% of the 1,385,159,111 bp assembly. The distribution of scaffold lengths is shown in dark grey with the plot radius scaled to the longest scaffold present in the assembly (100,421,371 bp, shown in red). Orange and pale-orange arcs show the N50 and N90 scaffold lengths (62,494,563 and 45,303,176 bp), respectively. The pale grey spiral shows the cumulative scaffold count on a log scale with white scale lines showing successive orders of magnitude. The blue and pale-blue area around the outside of the plot shows the distribution of GC, AT and N percentages in the same bins as the inner plot. A summary of complete, fragmented, duplicated and missing BUSCO genes in the eudicots_odb10 set is shown in the top right. An interactive version of this figure is available at https://blobtoolkit.genomehubs.org/view/CAUVWQ01/dataset/CAUVWQ01/snail.

**Figure 3.**
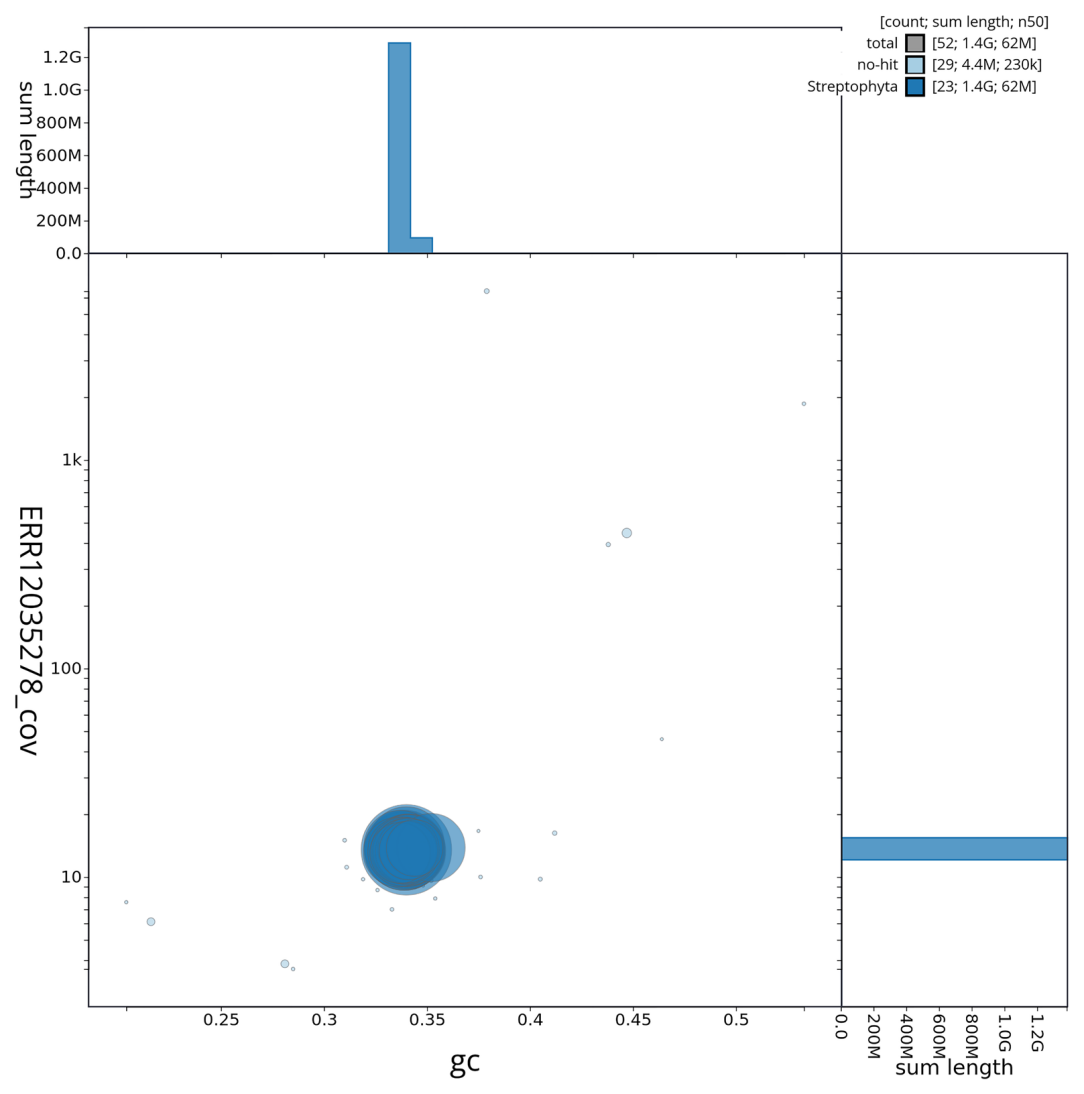
Genome assembly of *Ligustrum vulgare*, daLigVulg1.1: Blob plot of base coverage in ERR12035278 against GC proportion for sequences in the assembly. Sequences are coloured by phylum. Circles are sized in proportion to sequence length. Histograms show the distribution of sequence length sum along each axis. An interactive version of this figure is available at https://blobtoolkit.genomehubs.org/view/CAUVWQ01/dataset/CAUVWQ01/blob.

**Figure 4.**
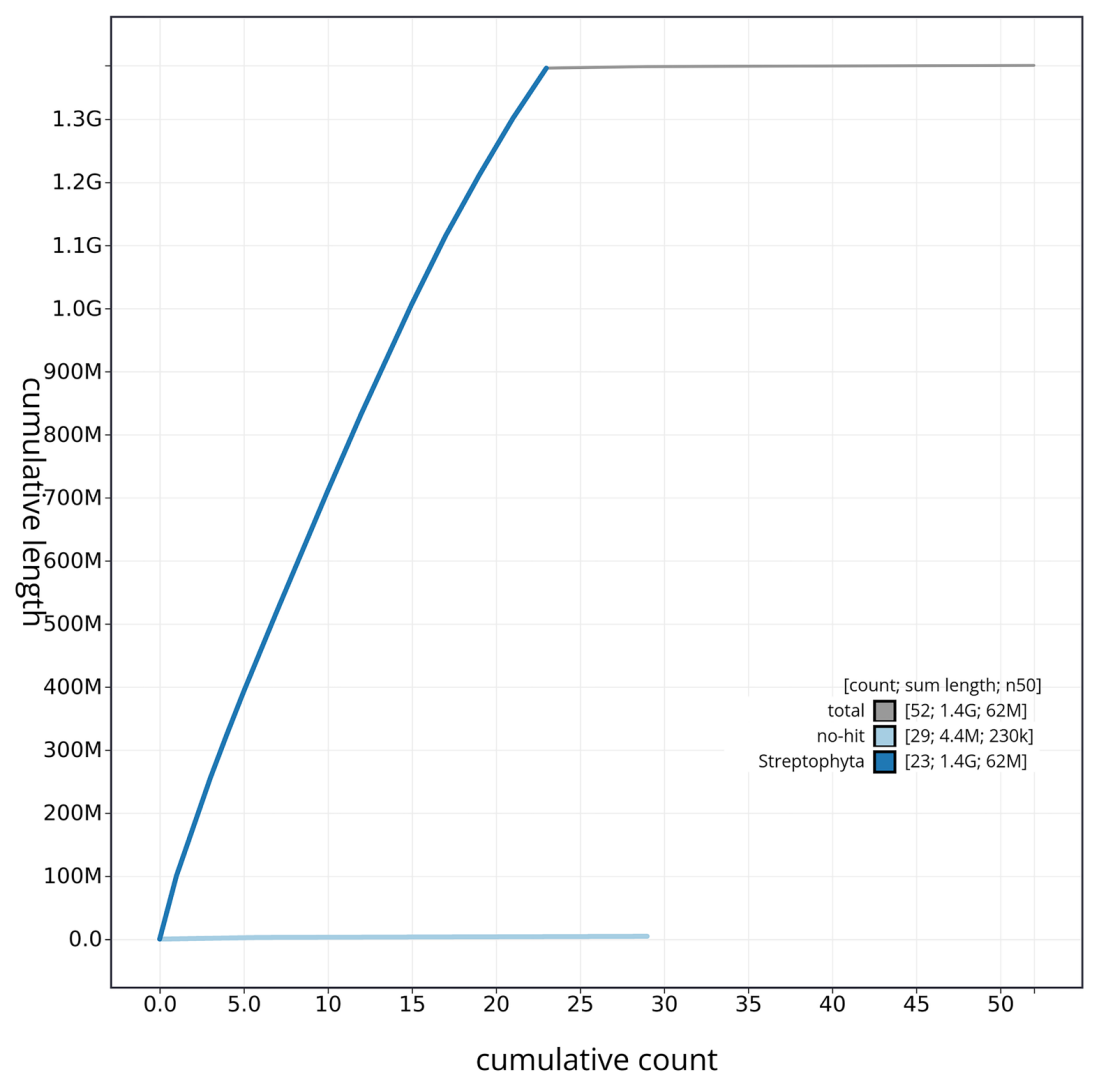
Genome assembly of *Ligustrum vulgare* daLigVulg1.1: BlobToolKit cumulative sequence plot. The grey line shows cumulative length for all sequences. Coloured lines show cumulative lengths of sequences assigned to each phylum using the buscogenes taxrule. An interactive version of this figure is available at https://blobtoolkit.genomehubs.org/view/CAUVWQ01/dataset/CAUVWQ01/cumulative.

**Figure 5.**
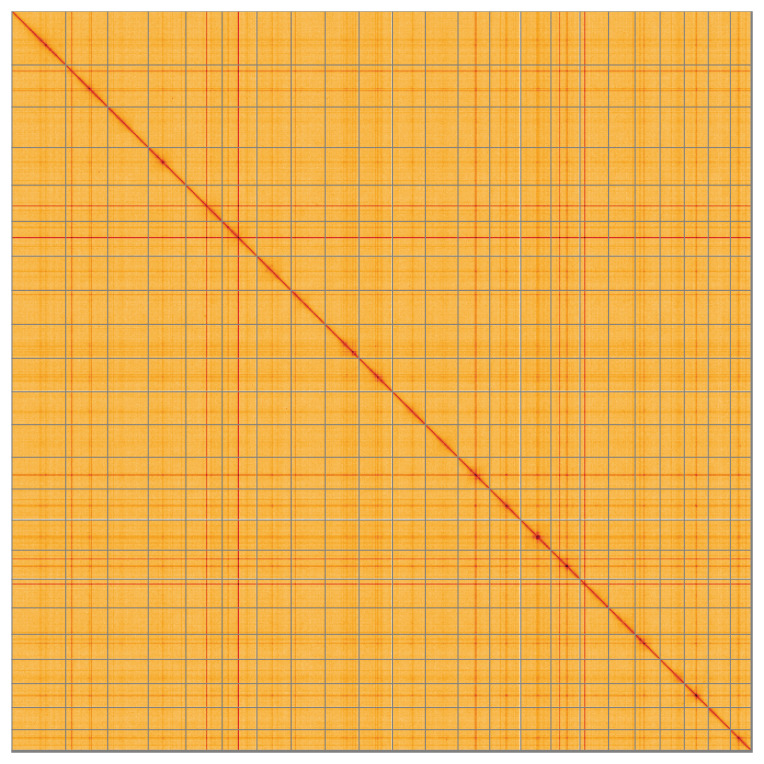
Genome assembly of *Ligustrum vulgare*, daLigVulg1.1: Hi-C contact map of the daLigVulg1.1 assembly, visualised using HiGlass. Chromosomes are shown in order of size from left to right and top to bottom. An interactive version of this figure may be viewed at https://genome-note-higlass.tol.sanger.ac.uk/l/?d=LiFrhIQ8RmSFRX2IFLesUw.

Manual assembly curation corrected 48 missing joins or mis-joins, reducing the scaffold number by 32.47%, and increasing the scaffold N50 by 1.25%. The final assembly has a total length of 1,384.00 Mb in 49 sequence scaffolds, with 181 gaps and a scaffold N50 of 62.5 Mb ( Table 2) The snail plot in Figure 2 provides a summary of the assembly statistics, while the distribution of assembly scaffolds on GC proportion and coverage is shown in Figure 3. The cumulative assembly plot in Figure 4 shows curves for subsets of scaffolds assigned to different phyla. Most (99.68%) of the assembly sequence was assigned to 23 chromosomal-level scaffolds. Chromosome-scale scaffolds confirmed by the Hi-C data are named in order of size ( Figure 5; Table 3). There appears to be a heterozygous translocation between chromosomes 20 and 21. While not fully phased, the assembly deposited is of one haplotype. Contigs corresponding to an alternate haplotype have also been deposited. The mitochondrial and plastid genomes were also assembled and can be found as contigs within the multifasta file of the genome submission.

**Table 2.** Genome assembly data for *Ligustrum vulgare*, daLigVulg1.1.

Genome assembly		
Assembly name	daLigVulg1.1	
Assembly accession	GCA_963555705.1	
*Accession of alternate haplotype*	*GCA_963555715.1*	
Span (Mb)	1,384.00	
Number of contigs	233	
Contig N50 length (Mb)	11.4	
Number of scaffolds	49	
Scaffold N50 length (Mb)	62.5	
Longest scaffold (Mb)	100.42	
Assembly metrics *		*Benchmark*
Consensus quality (QV)	62.2	*≥ 50*
*k*-mer completeness	Primary: 87.81%; alternate: 84.33%; combined: 97.76%	*≥ 95%*
BUSCO **	C:98.2%[S:85.7%,D:12.6%],F:0.7%,M:1.1%,n:2,326	*C ≥ 95%*
Percentage of assembly mapped to chromosomes	99.68%	*≥ 95%*
Organelles	Mitochondrial genome: 844.62 and 118.10 kb; plastid genome: 161.82 kb	*complete single alleles*
Genome annotation at Ensembl		
Number of protein-coding genes	31,482	
Number of non-coding genes	18,273	
Number of gene transcripts	62,120	

* Assembly metric benchmarks are adapted from column VGP-2020 of “Table 1: Proposed standards and metrics for defining genome assembly quality” from Rhie *et al.* (2021). ** BUSCO scores based on the eudicots_odb10 BUSCO set using version 5.4.3. C = complete [S = single copy, D = duplicated], F = fragmented, M = missing, n = number of orthologues in comparison. A full set of BUSCO scores is available at https://blobtoolkit.genomehubs.org/view/CAUVWQ01/dataset/CAUVWQ01/busco.

**Table 3.** Chromosomal pseudomolecules in the genome assembly of *Ligustrum vulgare*, daLigVulg1.

INSDC accession	Name	Length (Mb)	GC%
OY743168.1	1	100.42	34.0
OY743169.1	2	78.49	34.0
OY743170.1	3	75.69	34.0
OY743171.1	4	70.29	34.0
OY743172.1	5	67.64	34.0
OY743173.1	6	65.07	34.0
OY743174.1	7	63.98	33.5
OY743175.1	8	63.52	34.0
OY743176.1	9	63.26	34.0
OY743177.1	10	62.49	34.0
OY743178.1	11	61.41	34.0
OY743179.1	12	60.97	34.0
OY743180.1	13	59.27	33.5
OY743181.1	14	58.05	34.0
OY743182.1	15	56.32	35.0
OY743183.1	16	54.17	34.0
OY743184.1	17	53.49	34.0
OY743185.1	18	49.47	34.0
OY743186.1	19	46.75	34.0
OY743187.1	20	45.3	34.0
OY743188.1	21	44.63	33.5
OY743189.1	22	41.39	34.0
OY743190.1	23	38.65	34.5
OY743193.1	Pltd	0.16	38.0
OY743191.1	MT1	0.84	44.5
OY743192.1	MT2	0.12	44.0

The estimated Quality Value (QV) of the final assembly is 62.2 with *k*-mer completeness of 97.76% (combined primary and alternate). The primary assembly has a BUSCO v5.4.3 completeness of 98.2% (single = 85.7%, duplicated = 12.6%), using the eudicots_odb10 reference set ( *n* = 2,326).

## Genome annotation report

The *Ligustrum vulgare* genome assembly (GCA_963555705.1) was annotated at the European Bioinformatics Institute (EBI) on Ensembl Rapid Release. The resulting annotation includes 62,120 transcribed mRNAs from 31,482 protein-coding and 18,273 non-coding genes ( Table 2; https://rapid.ensembl.org/Ligustrum_vulgare_GCA_963555705.1/Info/Index). The average transcript length is 3,206.44. There are 1.25 coding transcripts per gene and 4.31 exons per transcript.

## Methods

### Sample acquisition, DNA barcoding and genome size estimation

Leaves from a *Ligustrum vulgare* shrub (specimen ID EDTOL00064, ToLID daLigVulg1) were collected from cultivated material at the Royal Botanic Garden Edinburgh (Inverleith), Midlothian, Scotland, UK (latitude 55.97, longitude –3.21), from a plant that was originally wild-collected by William Tait in 1984, from Epsom Downs, Surrey, England. Samples were collected on 2020-08-11 and on 2022-08-08. The samples were taken by Zoë Goodwin, David Bell and Vladimir Krivtsov (Royal Botanic Garden Edinburgh) and preserved by snap freezing in liquid nitrogen. The herbarium voucher from the sequenced plant is kept at the Royal Botanic Garden Edinburgh (E) https://data.rbge.org.uk/herb/E01358645.

The initial species identification was verified by an additional DNA barcoding process according to the framework developed by Twyford *et al*. (2024). Part of the plant specimen was preserved in silica gel desiccant. A DNA extraction from the dried plant was amplified by PCR for standard barcode markers, with the amplicons sequenced and compared to public sequence databases including GenBank and the Barcode of Life Database (BOLD). The barcode sequences for this specimen are openly available on BOLD ( Ratnasingham & Hebert, 2007). Following whole genome sequence generation, DNA barcodes were also used alongside the initial barcoding data for sample tracking through the genome production pipeline at the Wellcome Sanger Institute ( Twyford *et al.*, 2024). The standard operating procedures for the Darwin Tree of Life barcoding have been deposited on protocols.io ( Beasley *et al.*, 2023).

The genome size was estimated by flow cytometry using the fluorochrome propidium iodide and following the ‘one-step’ method as outlined in Pellicer *et al.* (2021). For this species, the General Purpose Buffer (GPB) supplemented with 3% PVP and 0.08% (v/v) beta-mercaptoethanol was used for isolation of nuclei ( Loureiro *et al.*, 2007), and the internal calibration standard was *Petroselinum crispum* ‘Champion Moss Curled’ with an assumed 1C-value of 2,200 Mb ( Obermayer *et al.*, 2002).

### Nucleic acid extraction

The workflow for high molecular weight (HMW) DNA extraction at the Wellcome Sanger Institute (WSI) Tree of Life Core Laboratory includes a sequence of core procedures: sample preparation; sample homogenisation, DNA extraction, fragmentation, and clean-up. In sample preparation, the daLigVulg1 sample was weighed and dissected on dry ice ( Jay *et al.*, 2023). For sample homogenisation, leaf tissue was cryogenically disrupted using the Covaris cryoPREP ^®^ Automated Dry Pulverizer ( Narváez-Gómez *et al.*, 2023). HMW DNA was extracted using the Automated Plant MagAttract v2 protocol ( Todorovic *et al.*, 2023). HMW DNA was sheared into an average fragment size of 12–20 kb in a Megaruptor 3 system ( Bates *et al.*, 2023). Sheared DNA was purified by solid-phase reversible immobilisation ( Strickland *et al.*, 2023): in brief, the method employs a 1.8X ratio of AMPure PB beads to sample to eliminate shorter fragments and concentrate the DNA. The concentration of the sheared and purified DNA was assessed using a Nanodrop spectrophotometer and Qubit Fluorometer and Qubit dsDNA High Sensitivity Assay kit. Fragment size distribution was evaluated by running the sample on the FemtoPulse system.

RNA was extracted from leaf tissue of daLigVulg1 in the Tree of Life Laboratory at the WSI using the RNA Extraction: Automated MagMax™ *mir*Vana protocol ( do Amaral *et al.*, 2023). The RNA concentration was assessed using a Nanodrop spectrophotometer and a Qubit Fluorometer using the Qubit RNA Broad-Range Assay kit. Analysis of the integrity of the RNA was done using the Agilent RNA 6000 Pico Kit and Eukaryotic Total RNA assay.

### Sequencing

Pacific Biosciences HiFi circular consensus DNA sequencing libraries were constructed according to the manufacturers’ instructions. Poly(A) RNA-Seq libraries were constructed using the NEB Ultra II RNA Library Prep kit. DNA and RNA sequencing was performed by the Scientific Operations core at the WSI on Pacific Biosciences Sequel IIe (HiFi) and Illumina NovaSeq 6000 (RNA-Seq) instruments. Hi-C data were also generated from leaf tissue of daLigVulg1 using the Arima-HiC v2 kit. The Hi-C sequencing was performed using paired-end sequencing with a read length of 150 bp on the Illumina NovaSeq 6000 instrument.

### Genome assembly, curation and evaluation

***Assembly***

The original assembly of HiFi reads was performed using Hifiasm ( Cheng *et al.*, 2021) with the --primary option. Haplotypic duplications were identified and removed with purge_dups ( Guan *et al.*, 2020). Hi-C reads were further mapped with bwa-mem2 ( Vasimuddin *et al.*, 2019) to the primary contigs, which were further scaffolded using the provided Hi-C data ( Rao *et al.*, 2014) in YaHS ( Zhou *et al.*, 2023) using the --break option. Scaffolded assemblies were evaluated using Gfastats ( Formenti *et al.*, 2022), BUSCO ( Manni *et al.*, 2021) and MERQURY.FK ( Rhie *et al.*, 2020). The organelle genomes were assembled using OATK ( Zhou, 2023).

***Curation***

The assembly was decontaminated using the Assembly Screen for Cobionts and Contaminants (ASCC) pipeline (article in preparation). Manual curation was primarily conducted using PretextView ( Harry, 2022), with additional insights provided by JBrowse2 ( Diesh *et al.*, 2023) and HiGlass ( Kerpedjiev *et al.*, 2018). Scaffolds were visually inspected and corrected as described by Howe *et al*. (2021). Any identified contamination, missed joins, and mis-joins were corrected, and duplicate sequences were tagged and removed. The process is documented at https://gitlab.com/wtsi-grit/rapid-curation (article in preparation).

***Evaluation of assembly quality***

The Merqury.FK tool ( Rhie *et al.*, 2020), run in a Singularity container ( Kurtzer *et al.*, 2017), was used to evaluate *k*-mer completeness and assembly quality for the primary and alternate haplotypes using the *k*-mer databases ( *k* = 31) computed prior to genome assembly. The analysis outputs included assembly QV scores and completeness statistics.

A Hi-C contact map was produced for the final version of the assembly. The Hi-C reads were aligned using bwa-mem2 ( Vasimuddin *et al.*, 2019) and the alignment files were combined using SAMtools ( Danecek *et al.*, 2021). The Hi-C alignments were converted into a contact map using BEDTools ( Quinlan & Hall, 2010) and the Cooler tool suite ( Abdennur & Mirny, 2020). The contact map was visualised in HiGlass ( Kerpedjiev *et al.*, 2018).

Table 4 contains a list of relevant software tool versions and sources.

**Table 4.** Software tools: versions and sources.

Software tool	Version	Source
BEDTools	2.30.0	https://github.com/arq5x/bedtools2
Blast	2.14.0	ftp://ftp.ncbi.nlm.nih.gov/blast/executables/blast+/
BlobToolKit	4.3.7	https://github.com/blobtoolkit/blobtoolkit
BUSCO	5.4.3 and 5.5.0	https://gitlab.com/ezlab/busco
bwa-mem2	2.2.1	https://github.com/bwa-mem2/bwa-mem2
Cooler	0.8.11	https://github.com/open2c/cooler
DIAMOND	2.1.8	https://github.com/bbuchfink/diamond
fasta_windows	0.2.4	https://github.com/tolkit/fasta_windows
FastK	427104ea91c78c3b8b8b49f1a7d6bbeaa869ba1c	https://github.com/thegenemyers/FASTK
Gfastats	1.3.6	https://github.com/vgl-hub/gfastats
GoaT CLI	0.2.5	https://github.com/genomehubs/goat-cli
Hifiasm	0.16.1-r375	https://github.com/chhylp123/hifiasm
HiGlass	44086069ee7d4d3f6f3f0012569789ec138f42b84aa44357826c0b6753eb28de	https://github.com/higlass/higlass
Merqury.FK	d00d98157618f4e8d1a9190026b19b471055b22e	https://github.com/thegenemyers/MERQURY.FK
MitoHiFi	2	https://github.com/marcelauliano/MitoHiFi
MultiQC	1.14, 1.17, and 1.18	https://github.com/MultiQC/MultiQC
NCBI Datasets	15.12.0	https://github.com/ncbi/datasets
Nextflow	23.04.0-5857	https://github.com/nextflow-io/nextflow
PretextView	0.2.5	https://github.com/sanger-tol/PretextView
OATK	0.9	https://github.com/c-zhou/oatk
purge_dups	1.2.3	https://github.com/dfguan/purge_dups
samtools	1.16.1, 1.17, and 1.18	https://github.com/samtools/samtools
Seqtk	1.3	https://github.com/lh3/seqtk
Singularity	3.9.0	https://github.com/sylabs/singularity
YaHS	1.1a.2	https://github.com/c-zhou/yahs

### Genome annotation

The Ensembl Genebuild annotation system ( Aken *et al.*, 2016) was used to generate annotation for the *Ligustrum vulgare* assembly (GCA_963555705.1) in Ensembl Rapid Release at the EBI. Annotation was created primarily through alignment of transcriptomic data to the genome, with gap filling via protein-to-genome alignments of a select set of proteins from UniProt ( UniProt Consortium, 2019).

### Wellcome Sanger Institute – Legal and Governance

The materials that have contributed to this genome note have been supplied by a Darwin Tree of Life Partner. The submission of materials by a Darwin Tree of Life Partner is subject to the **‘Darwin Tree of Life Project Sampling Code of Practice’**, which can be found in full on the Darwin Tree of Life website here. By agreeing with and signing up to the Sampling Code of Practice, the Darwin Tree of Life Partner agrees they will meet the legal and ethical requirements and standards set out within this document in respect of all samples acquired for, and supplied to, the Darwin Tree of Life Project.

Further, the Wellcome Sanger Institute employs a process whereby due diligence is carried out proportionate to the nature of the materials themselves, and the circumstances under which they have been/are to be collected and provided for use. The purpose of this is to address and mitigate any potential legal and/or ethical implications of receipt and use of the materials as part of the research project, and to ensure that in doing so we align with best practice wherever possible. The overarching areas of consideration are:

•   Ethical review of provenance and sourcing of the material

•   Legality of collection, transfer and use (national and international) 

Each transfer of samples is further undertaken according to a Research Collaboration Agreement or Material Transfer Agreement entered into by the Darwin Tree of Life Partner, Genome Research Limited (operating as the Wellcome Sanger Institute), and in some circumstances other Darwin Tree of Life collaborators.

## Data Availability

European Nucleotide Archive: *Ligustrum vulgare* (common privet). Accession number PRJEB65698; https://identifiers.org/ena.embl/PRJEB65698. The genome sequence is released openly for reuse. The *Ligustrum vulgare* genome sequencing initiative is part of the Darwin Tree of Life (DToL) project. All raw sequence data and the assembly have been deposited in INSDC databases. Raw data and assembly accession identifiers are reported in Table 1.

## Author information

Members of the Royal Botanic Garden Edinburgh Genome Acquisition Lab are listed here: https://doi.org/10.5281/zenodo.4786682.

Members of the Plant Genome Sizing collective are listed here: https://doi.org/10.5281/zenodo.7994306.

Members of the Darwin Tree of Life Barcoding collective are listed here: https://doi.org/10.5281/zenodo.12158331

Members of the Wellcome Sanger Institute Tree of Life Management, Samples and Laboratory team are listed here: https://doi.org/10.5281/zenodo.12162482.

Members of Wellcome Sanger Institute Scientific Operations: Sequencing Operations are listed here: https://doi.org/10.5281/zenodo.12165051.

Members of the Wellcome Sanger Institute Tree of Life Core Informatics team are listed here: https://doi.org/10.5281/zenodo.12160324.

Members of the Tree of Life Core Informatics collective are listed here: https://doi.org/10.5281/zenodo.12205391.

Members of the Darwin Tree of Life Consortium are listed here: https://doi.org/10.5281/zenodo.4783558.
