# Supplementary material for: The genome sequence of wild privet, Ligustrum vulgare L
Source: Wellcome Open Res. 2025 May 16;10:240. [Version 1] doi: 10.12688/wellcomeopenres.24023.1 (PMC12171723; doi:10.12688/wellcomeopenres.24023.1)
Supplement: Peer review history

# Reviewer report: Reviewer response for version 1

**DOI:** 10.21956/wellcomeopenres.26505.r124703

**Published:** 2025-06-16

**Authors:** Marco Pessoa-Filho

**Affiliations:** 1 Brazilian Agricultural Research Corporation, Brasília, Brazil

The data note describes the assembly and annotation of the genome of the wild privet ( *Ligustrum vulgare*). The authors used third-generation sequencing with PacBio HiFi reads assembled with Hifiasm. Using YAHS, the draft was scaffolded with Hi-C data generated with the Arima Hi-C kit. Manual curation was performed with PretextView following the Rapid Curation protocol of the Genome Reference Informatics Team at the Wellcome Sanger Institute. Annotation was performed with the Ensemble Genebuild annotation system.

 It is unclear why purge_dups was used after hifiasm, since hifiasm already includes a purging step based on a purge graph using read phasing, and not inexact contig alignment. The level of BUSCO duplication is also worth mentioning (12.6%) and not discussed. No details on the species' reproductive mode and heterozygosity level are presented (is it an outcrosser?). Results of a GeneScope analysis, which must have been performed, could enrich the report.

 Also, more recent versions of Hifiasm allow using Hi-C reads for haplotype phasing. 

 Details are lacking for the decontamination pipeline, which mentions an article in preparation. 

 The annotation pipeline is fully documented elsewhere. An estimate of BUSCO completeness or OMark proteome quality assessment would also enrich the report.

Are sufficient details of methods and materials provided to allow replication by others?

Partly

Is the rationale for creating the dataset(s) clearly described?

Yes

Are the datasets clearly presented in a useable and accessible format?

Yes

Are the protocols appropriate and is the work technically sound?

Yes

Reviewer Expertise:

Plant genomics, bioinformatics, and plant genetics.

I confirm that I have read this submission and believe that I have an appropriate level of expertise to confirm that it is of an acceptable scientific standard, however I have significant reservations, as outlined above.

# Reviewer report: Reviewer response for version 1

**DOI:** 10.21956/wellcomeopenres.26505.r124000

**Published:** 2025-06-13

**Authors:** Irene Julca

**Affiliations:** 1 University of Lausanne, Lausanne, Switzerland

Goodwin and collaborators present the nuclear and organellar genomes of *Ligustrum vulgare* (wild privet), along with their gene annotations. I believe this work represents a valuable resource for the plant research community, particularly for studies involving genome evolution, comparative genomics, and functional analysis.

 The manuscript is well written and clearly presented. The authors have provided detailed methods, and all relevant links and datasets (including those related to the figures) are appropriately included and accessible.

 My only comment concerns the gene annotation of the organellar genomes, which is not clearly described. It would be helpful if the authors could clarify whether the annotation of plastid and mitochondrial genomes was performed. I downloaded the gff file from Ensembl, but I couldn’t determine if any of the contigs correspond to the organellar genomes. It will be useful if the authors could provide more information on this point.

Are sufficient details of methods and materials provided to allow replication by others?

Yes

Is the rationale for creating the dataset(s) clearly described?

Yes

Are the datasets clearly presented in a useable and accessible format?

Yes

Are the protocols appropriate and is the work technically sound?

Yes

Reviewer Expertise:

Genome assembly and annotation, phylogenomics, transcriptomics, evolution

I confirm that I have read this submission and believe that I have an appropriate level of expertise to confirm that it is of an acceptable scientific standard.

# Reviewer report: Reviewer response for version 1

**DOI:** 10.21956/wellcomeopenres.26505.r124697

**Published:** 2025-06-06

**Authors:** Abhisek Chakraborty

**Affiliations:** 1 Johannes Gutenberg University of Mainz, Mainz, Germany

In this data note, the authors reported a high-quality genome assembly of *Ligustrum vulgare* and also performed the genome annotation. I have the following comments for this manuscript –

1. Please discuss the reason for the presence of >10% duplicated BUSCO genes in the genome assembly.
2. The genome annotation results should be mentioned in the manuscript in more details. For example, please report the whole genome repeat content and the BUSCO completeness of the coding gene set.
3. Among the 18,273 non-coding genes, what are the different categories (e.g., tRNA, rRNA, etc.)? How many organellar genes were identified in this species?
4. The assembly decontamination method using the Assembly Screen for Cobionts and Contaminants (ASCC) pipeline is not clear since the pipeline is not published yet.

Are sufficient details of methods and materials provided to allow replication by others?

Partly

Is the rationale for creating the dataset(s) clearly described?

Yes

Are the datasets clearly presented in a useable and accessible format?

Yes

Are the protocols appropriate and is the work technically sound?

Yes

Reviewer Expertise:

Genome Assembly, Evolutionary Genomics

I confirm that I have read this submission and believe that I have an appropriate level of expertise to confirm that it is of an acceptable scientific standard, however I have significant reservations, as outlined above.
